# Enhanced Mechanical Properties of Lightweight Ti_65_(AlCrNbV)_35_ Medium-Entropy Alloys via Microstructure Modification Through Minor B Doping

**DOI:** 10.3390/ma18102219

**Published:** 2025-05-11

**Authors:** Po-Sung Chen, Bao-Teng Kuo, Pei-Hua Tsai, Jason Shian-Ching Jang, Chih-Yen Chen, I-Yu Tsao

**Affiliations:** 1 Institute of Materials Science and Engineering, National Central University, Taoyuan 320, Taiwan; 2 Department of Mechanical Engineering, National Central University, Taoyuan 320, Taiwan; 3 Department of Electrophysics, National Yang Ming Chiao Tung University, Hsinchu 300, Taiwan

**Keywords:** lightweight medium-entropy alloys, minor element doping, nonequiatomic, thermomechanical treatment

## Abstract

Because of their low density and excellent material properties, lightweight Ti-rich medium-entropy alloys (MEAs) have great potential for application in the aerospace and automotive industries. This study investigated the effects of B doping on the microstructure and mechanical properties of a (Ti_65_(AlCrNbV)_35_)_100−x_B_x_ alloy series. The mechanical properties of the alloys were then enhanced through thermomechanical treatment, and the strengthening mechanism was explored by characterizing the alloys’ microstructure and mechanical properties. X-ray diffraction revealed that the (Ti_65_(AlCrNbV)_35_)_100−x_B_x_ alloys retained their body-centered cubic structure. However, the addition of B resulted in a rightward shift in the diffraction peaks due to B having a smaller atomic radius compared with the other constituent elements. Weak diffraction peaks corresponding to TiB were discovered in the diffraction patterns for the alloys with 0.4 or 0.6% B content (named B0.4 and B0.6, respectively). The hardness of the homogenized alloys was increased from 321 Hv for the base alloy (B0) to 378 Hv for B0.6. In tensile testing, the homogenized alloy with 0.2% B content (B0.2) exhibited a yield strength of 1054 MPa and 21% elongation, which represented 17% greater strength compared with B0. Conversely, the mechanical properties of B0.4 and B0.6 were poorer due to precipitation at grain boundaries. After thermomechanical treatment, the alloys’ strength and hardness increased with increasing B content despite various heat treatment conditions. The recrystallization behavior of the alloys tended to be delayed by B doping, resulting in an increase in the recrystallization temperature. After recrystallization at 900 °C, the elongation of B0, B0.1, and B0.2 exceeded 20%. Of the (Ti_65_(AlCrNbV)_35_)_100−x_B_x_ alloys in the series, B0.2 presents the optimal combination of favorable yield strength and ductility (1275 MPa and 10%, respectively).

## 1. Introduction

Since the beginning of industrialization, commercial aviation has facilitated a range of human activities. Aircraft are being operated in increasingly challenging environments and must be strong [1]. Traditional lightweight aircraft materials have limitations. For example, these materials are weak and have poor temperature resistance [2]. Steel has outstanding mechanical properties but considerable mass. Aircraft made of steel would consume more fuel than an aircraft made from light materials would. Aircraft made of steel would also be difficult to control, leading to higher risks [3]. Novel materials with high specific strength are required in the aerospace and energy industries.

Medium-entropy alloys (MEAs) may be used to develop high-performance, low-density materials. MEAs comprise several principal elements [4,5]. This characteristic will not only avoid the complex phases generated but also expand the range of alloy design [6,7]. Therefore, the MEAs possess excellent material properties and flexibility of element modification which exhibit huge potential for high performance material exploration [8]. In addition, the mechanical properties of MEAs can be improved using solid solution strengthening [9]. Fine-tuning the substitutional or interstitial elements can distort the lattice of the material and thereby impede the propagation of dislocations, enhancing the mechanical properties of the alloy [10,11]. Moreover, the mechanical performance of MEAs can be enhanced using grain-boundary strengthening [12]. With the minor addition of a grain refiner such as boron, the grain size will be reduced and the strength will be improved [13,14,15].

The mechanical properties of alloys can also be improved using thermomechanical treatment (TMT) [16]. Hot-rolling processes can refine the grain size and eliminate the defects in as-cast alloys. Cold-rolling processes can store strain energy, promoting recrystallization behavior in annealing [17,18]. In addition, the rapid thermal process (RTP) can also improve the material properties of an alloy. Rapid annealing involves high temperatures and short annealing periods which can provide high driving forces, leading to the nucleation of more nuclei and preventing the coarsening of recrystallized grains [19]. Rapid annealing effectively reduces grain size in a recrystallized alloy and enhances the mechanical properties of alloys through strengthening of grain boundaries [20].

The quaternary Ti65 alloy possesses a simple structure and outstanding tensile mechanical properties [21]; therefore, it has been further developed to the quinary and senary lightweight MEAs [22,23,24,25]. On the other hand, the thermo-mechanical treatment process would also be used to further optimize the microstructure and enhance the mechanical properties of the MEAs [19,23,24,25,26]. In this study, a series of lightweight Ti-rich MEAs were doped with a small amount of B to modify the MEAs’ microstructure and improve their mechanical properties. Adding B not only reduced the size of grains but also distorted the lattice of the alloy to improve its mechanical properties. In addition, TMT and rapid annealing were conducted to further enhance the microstructure and mechanical properties of the MEAs.

## 2. Materials and Methods

### 2.1. Materials

Based on the previous study [23], a series of novel (Ti_65_(AlCrNbV)_35_)_100−x_B_x_ alloys (where x = 0, 0.1, 0.2, 0.4, or 0.6, with the corresponding MEAs named B0, B0.1, B0.2, B0.4, and B0.6) were mircoalloyed using high-purity Ti (99.99%), Al (99.99%), Cr (99.99%), Nb (99.95%), V (99.9%), and B (99.5%) raw materials. An arc-melting furnace was employed to prepare the alloys in argon atmosphere to prevent oxidation. The alloys were remelted four times to ensure their homogeneity. Ingots of size 35 mm × 20 mm × 14 mm were then fabricated through drop casting. Subsequently, the ingots were homogenized at 1000 °C for 6 h in a high-vacuum atmosphere (10^−6^ mbar), after which they were water-quenched.

### 2.2. Processing

To optimize the morphology and mechanical properties, the MEAs were subjected to TMT and rapid annealing. The homogenized ingots were hot-rolled at 1000 °C to achieve 50% thickness reduction and then cold-rolled at room temperature to achieve 80% thickness reduction (this process is abbreviated to HR50CR80). Subsequently, the deformed samples were subjected to rapid annealing (heating rate = 15 °C/s) at one of three temperatures (700, 800, or 900 °C) in a high-vacuum atmosphere (10^−6^ mbar), after which they were water-quenched.

### 2.3. Microstructure Characterization

MEA density was measured in accordance with Archimedes’ principle. MEA structure was analyzed using an X-ray diffractor (D2, Bruker, Billerica, MA, USA) with Cu-Kα radiation. Each sample was ground using pieces of silicon carbide sandpaper with grit sizes ranging from #80 to #2000. MEA morphology was observed using an optical microscope (BX51M, Olympus, Tokyo, Japan), an electron backscatter diffractor (HKL Channel 5, Oxford Instruments, Hobro, Denmark), and a transmission electron microscope (JEM2000FXII & JEM-2100, JEOL, Tokyo, Japan). For optical microscopy, the samples were polished using sequential suspensions of Al_2_O_3_ particles with sizes of 1, 0.3, and 0.05 µm. For electron backscatter diffraction characterization, the samples were polished using an electropolishing machine. For transmission electron microscopy analysis, each specimen was prepared using a focused ion beam–scanning electron microscope (Versa 3D, FEI, Hillsboro, OR, USA).

### 2.4. Mechanical Properties Test

The hardness of the alloys was measured using a Vickers hardness tester (HV-115, Mitutoyo, Kawasaki, Japan) with application of a 5 kg load for 10 s. The alloys’ tensile mechanical properties were tested using a universal testing machine (HT9102, Hung Ta, Taichung, Taiwan) under quasistatic loading and a strain rate of 1 × 10^−4^/s. The gauge dimensions of the tensile testing specimens were 5 mm (length) × 2 mm (width) × 1.5 mm (thickness).

## 3. Results and Discussion

On the basis of our previous studied quinary lightweight Ti_65_(AlCrNbV)_35_ MEA, a series of lightweight (Ti_65_(AlCrNbV)_35_)_100−x_B_x_ MEAs were designed (Table 1). The configuration entropy of the alloys was 9.42–9.66 kJ/mol. The entropy of the MEAs increased slightly with an increase in the degree of B doping. The MEAs had similar atomic size differences (δr), 4.78–8.71, and this difference was positively correlated with the degree of B doping.

### 3.1. Properties of Homogenized (Ti_65_(AlCrNbV)_35_)_100−x_B_x_ MEAs

**Figure 1:**
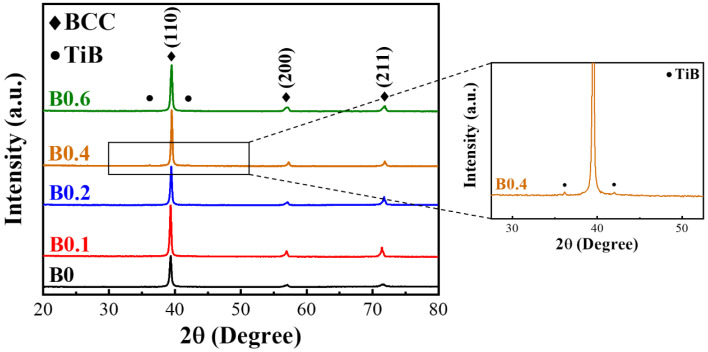
X-ray diffraction patterns of homogenized (Ti_65_(AlCrNbV)_35_)_100−x_B_x_ medium-entropy alloys (MEAs).

**Figure 2:**
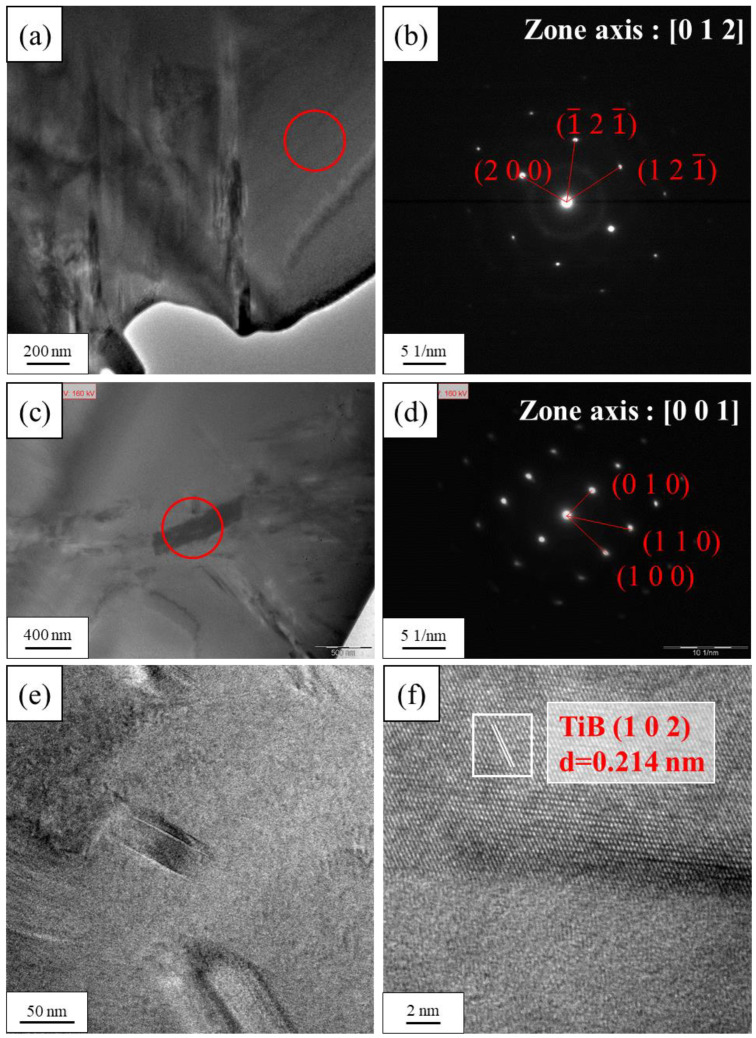
Transmission electron microscopy images of homogenized B0.4 MEA. (**a**,**b**) Bright-field and selected area diffraction pattern of matrix; (**c**,**d**) bright-field and selected area diffraction pattern of TiB precipitate; and (**e**,**f**) lattice image of TiB precipitate.

The measured densities of the alloys were all similar at approximately 5.10 g/cm^3^, which agreed with the theoretical densities calculated using the mixing rule (Table 2). The MEAs presented the characteristic peaks of the body-centered cubic phase in the X-ray diffraction analysis (Figure 1). While the concentration of B increased to 0.4%, the weak diffraction peak could also be noticed. In addition, the main diffraction peaks of (1 1 0) shifted slightly to the right with increasing B doping due to lattice distortion caused by the atomic size difference δ that resulted from the small atomic size of B, while the size of B was 31–52 pm smaller than that of the other elements in the alloy. Moreover, the precipitate of B0.4 and B0.6 alloys was identified as TiB intermetallic phase through TEM observation (Figure 2) [27]. This indicated that the addition of excessive B resulted in the formation of a detectable amount of TiB compound after homogenization.

**Figure 3:**
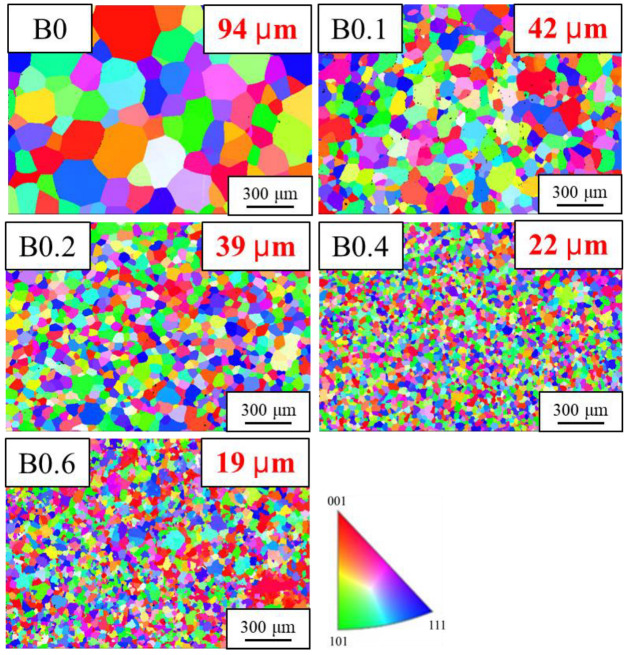
Electron backscatter diffraction images of homogenized (Ti_65_(AlCrNbV)_35_)_100−x_B_x_ MEAs.

**Figure 4:**
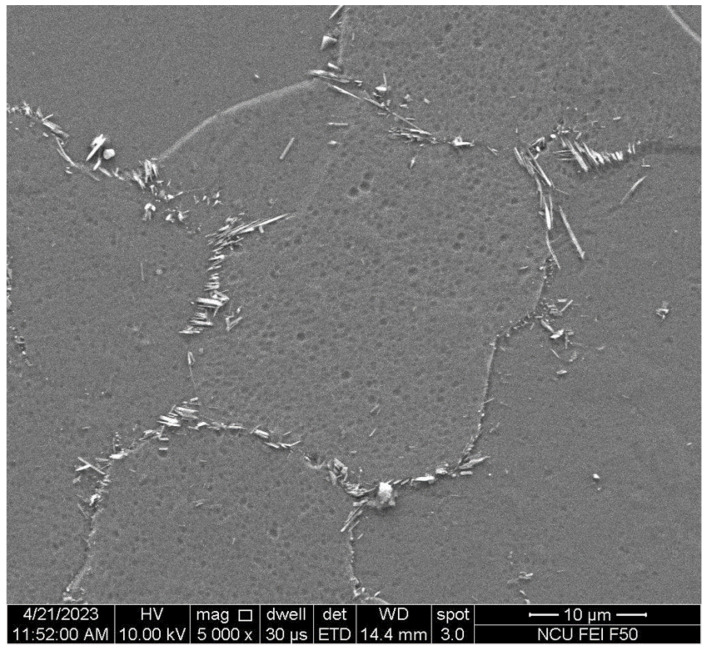
Scanning electron microscopy images of homogenized B0.4 MEA.

The results of the metallographic observation revealed that B doping indeed resulted in smaller grains in the homogenized MEAs (Figure 3). The grain size was 94 μm for B0 to 19 μm for B0.6. This agrees with the reported effect of B doping on grain refinement [15]. TiB has a very large negative heat of mixing (−84 kJ/mole) [28]. Therefore, TiB compounds form at first during the solidification process and act as seeds to provide more nucleation sites for heterogeneous nucleation, resulting in smaller grains. When B doping was excessive, B segregated at the grain boundary and formed TiB particles, which inhibited grain growth and embrittled the alloy due to the concentration of stress in the hard plate-like TiB precipitates along the grain boundaries (Figure 4).

**Figure 5:**
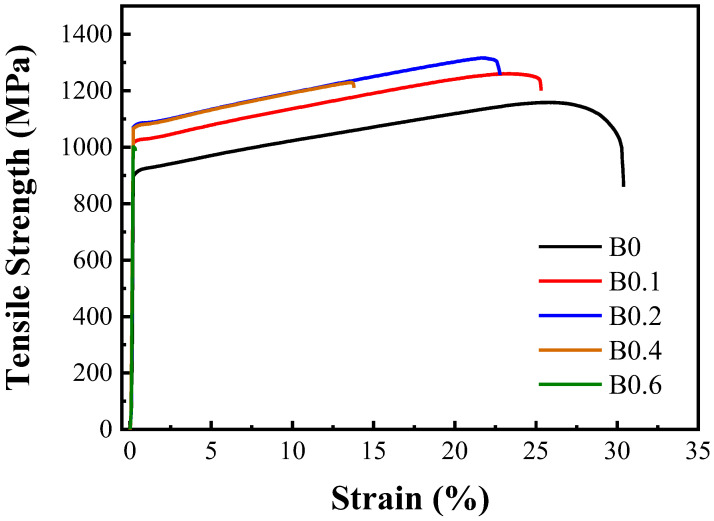
Stress–strain curve of homogenized (Ti_65_(AlCrNbV)_35_)_100−x_B_x_ MEAs.

The results of the hardness and tensile tests of the homogenized MEAs revealed that B doping increased both the hardness and yield strength (Table 3). The increases in hardness (approximately 9–11%) and yield strength (approximately 11–18%) were presumed to be attributable to smaller grains in the MEA ingots and the effect of solid-solution strengthening achieved through the addition of B. However, the tensile ductility of the MEAs decreased with an increase in the degree of B doping. B0.4 had low ductility, and B0.6 even exhibited embrittlement. This indicated that an alloy containing excessive B was embrittled due to the formation of plate-like TiB precipitates along grain boundaries (Figure 5).

To enhance the mechanical properties of the MEAs, their microstructures (e.g., fine grains and heterostructure) had to be modified using TMT.

### 3.2. Performance of B-Doped MEAs After TMT Processing

**Figure 6:**
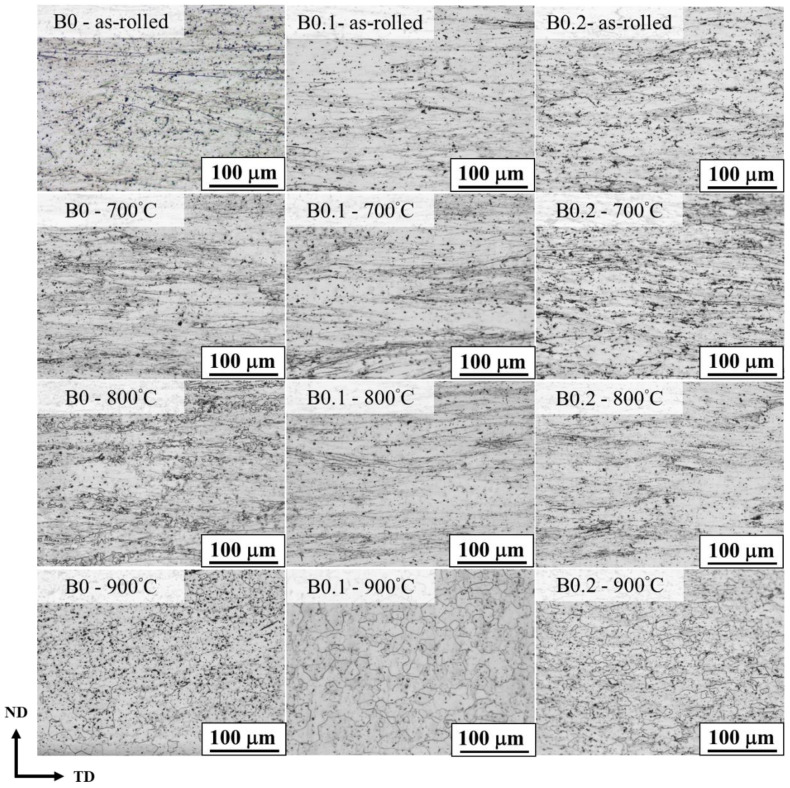
Optical microscopy images of (Ti_65_(AlCrNbV)_35_)_100−x_B_x_ MEAs after thermomechanical treatment.

The homogenized MEA ingots with high ductility—B0, B0.1, and B0.2—were further processed using TMT and rapid annealing; the samples were subjected to HR50CR80 processing and then rapidly annealed for 27, 38, or 70 s at a heating rate of 15 °C/s to reach the temperatures 700, 800, and 900 °C, respectively. The results of optical microscopy revealed that only B0 began to recrystallize (which occurred at a temperature of 800 °C); a small area of recrystallized grain can be seen in Figure 6. The other alloys only had a deformation band at a temperature of 800 °C. All alloys fully recrystallized at 900 °C (Figure 6). These results are attributable to the interstitial B atoms inhibiting the interface migration of nucleation sites during recrystallization, leading to higher recrystallization temperature [11,29,30]. The average grain size after recrystallization at 900 °C under a heating rate of 15 °C/s was discovered to decrease with increasing B doping; this size was 21.4 μm for B0 to 14.4 μm for B0.2. This finding was due to the delaying effect of B doping on recrystallization behavior, resulting in the average grain size at a given annealing temperature (e.g., 900 °C) being reduced.

**Figure 7:**
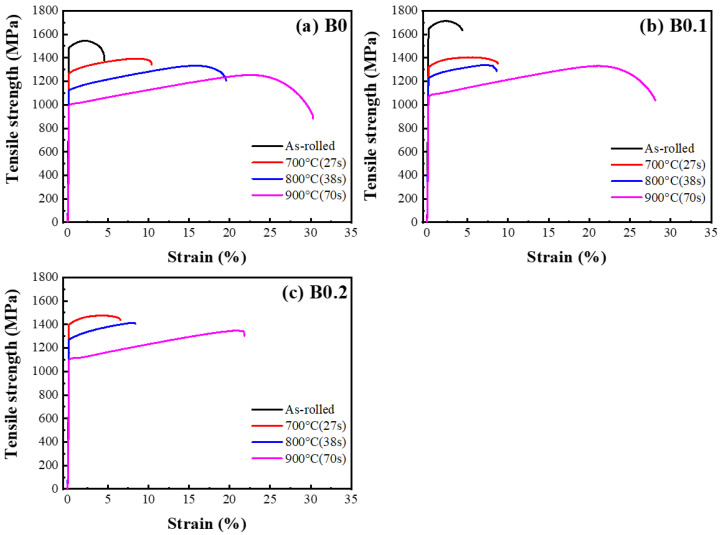
Mechanical tensile stress–strain curves of (Ti_65_(AlCrNbV)_35_)_100−x_B_x_ MEAs after thermomechanical treatment. (**a**) B0 MEA; (**b**) B0.1 MEA; and (**c**) B0.2 MEA.

Figure 7 and Table 4 and Table 5 show the results of the hardness and tensile testing conducted after the B0, B0.1, and B0.2 MEAs were subjected to TMT. The yield strengths of the as-rolled B0 and B0.1 samples were approximately 1480 and 1650 MPa, respectively, and these values were maintained under a plastic strain of approximately 4.5%. The as-rolled B0.2 sample exhibited brittleness and fractured before yielding due to its excessive hardness (B0.2 Hv 456 vs. B0.1 Hv 435). When the annealing time was increased and the heating rate was kept at 15 °C/s, the yield strengths of the B0, B0.1, and B0.2 alloys annealed for 27 s (the sample temperature reached 700 °C) decreased to approximately 1250, 1325, and 1350 MPa, respectively. Subsequently, the slope of the decrease in yield strength started to flatten when the annealing time increased. The yield strengths decreased to approximately 1090, 1200, and 1270 MPa for B0, B0.1, and B0.2, respectively, when annealing was performed for 38 s (the sample temperature reached 800 °C). The slightly descending slope of the change in yield strength was attributable to the existence of partially recrystallized fine grains coexisting with large residual strain area (Figure 6), which restricted dislocation and enhanced yield strength. On the other hand, the ductility of the samples increased to more than 10%. Furthermore, the yield strength of the samples of B0, B0.1, and B0.2 alloy that were annealed for 70 s (sample temperature reached 900 °C) decreased to around 1000 to 1100 MPa, indicating that the sample was almost fully recrystallized. By contrast, the ductility of this sample showed a significant increase of up to more than 25%.

**Figure 8:**
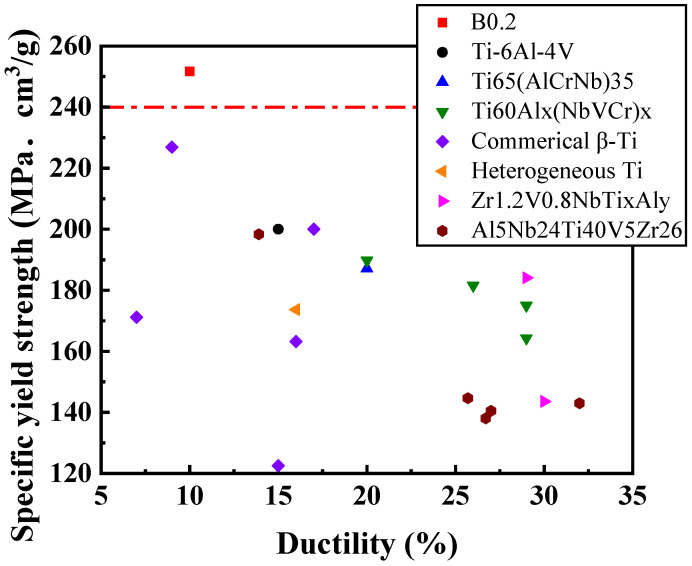
Specific yield strength and ductility comparison. Dashed line separates present work from other studies [20,21,22,31,32,33].

In physical metallurgy, grain refinement is an effective way of improving a material’s yield strength and ductility. In this study, the average grain size of the Ti_65_(AlCrNbV)_35_ base alloy was successfully reduced through minor B doping. During TMT, interstitial B atoms inhibit the interfacial migration of nucleation sites during recrystallization, delaying recrystallization and resulting in smaller grains at a given annealing temperature. Accordingly, of all the MEA samples in this study that were subjected to TMT, B0.1 and B0.2 exhibited excellent combinations of favorable yield strength (>1200 MPa) and ductility (>10%); notably, the B0.2 sample subjected to HR50CR80 processing and 38 s of annealing (800 °C) had yield strength of 1275 MPa and ductility of 10% (Figure 7 and Table 4). As shown in Figure 8, the B0.2 MEA after suitable TMT exhibited a specific yield strength of 250 MPa·cm^3^/g and a ductility of 10% (marked with a red square). These values are superior to those of commercial Ti alloys and some other lightweight HEAs and MEAs [20,21,22,31,32,33].

## 4. Conclusions

A series of lightweight (Ti_65_(AlCrNbV)_35_)_100−x_B_x_ MEAs were successfully prepared and subjected to TMT. The microstructure evolution and mechanical properties of the MEAs are summarized as follows:

1. X-ray diffraction analysis indicated that the homogenized (Ti_65_(AlCrNbV)_35_)_100−x_B_x_ alloys retained their body-centered cubic structure. However, when the B doping amount was 0.4%, weak diffraction peaks corresponding to TiB coexisted in the diffraction pattern.
2. Metallography revealed that B doping indeed reduced the grain size of the alloys. The post-homogenization grain size was 94 μm for B0, 42 μm for B0.1, 39 μm for B0.2, 22 μm for B0.4, and 19 μm for B0.6. Conversely, too much B resulted in the formation of TiB particles along the grain boundaries, considerably decreasing the ductility of the alloy.
3. The B0.2 homogenized MEA had a tensile yield strength of 1054 MPa and exhibited 21% elongation, which represents 17% higher strength than that of the base alloy. Conversely, the mechanical properties of B0.4 and B0.6 were poorer due to the formation of TiB precipitates at grain boundaries.
4. After TMT, the recrystallization behavior of the B-doped alloys tended to be delayed to a higher B addition, resulting in smaller grains on average at a given annealing temperature (e.g., 900 °C). The yield strength and hardness of the B-doped alloys increased with increasing B content.
5. Of all the MEA samples subjected to TMT in this study, B0.1 and B0.2 exhibited excellent combinations of yield strength (>1200 MPa) and ductility (>10%). Notably, the B0.2 MEA, after suitable TMT, exhibited a specific yield strength of 250 MPa·cm^3^/g and a ductility of 10%. These values are superior to those of commercial Ti alloys and some other lightweight alloys.

## Figures and Tables

**Table 1:** Parameters of (Ti_65_(AlCrNbV)_35_)_100−x_B_x_ medium-entropy alloys (MEAs).

Composition	ΔS (kJ·mol^−1^)	Δr (%)
B0	9.42	4.78
B0.1	9.47	5.63
B0.2	9.52	6.36
B0.4	9.59	7.63
B0.6	9.66	8.71

**Table 2:** Density of (Ti_65_(AlCrNbV)_35_)_100−x_B_x_ MEAs.

Composition	Theoretical Density (g/cm^3^)	Measured Density (g/cm^3^)	Error (%)
B0	5.101	5.091	0.19
B0.1	5.098	5.137	0.76
B0.2	5.095	5.066	0.56
B0.4	5.088	5.133	0.88
B0.6	5.082	5.043	0.76

**Table 3:** Hardness and tensile mechanical properties of homogenized (Ti_65_(AlCrNbV)_35_)_100−x_B_x_ MEAs.

Composition	Hardness (HV)	Yield Strength (MPa)	Ultimate Strength (MPa)	Ductility (%)
B0	321 ± 3	903 ± 9	1112 ± 34	27 ± 3
B0.1	356 ± 2	1011 ± 5	1232 ± 28	24 ± 1
B0.2	363 ± 3	1054 ± 17	1286 ± 31	21 ± 1
B0.4	367 ± 2	1066	1227	14
B0.6	378 ± 1	Rupture		
Ti-6Al-4V	318	885	985	15

**Table 4:** Vickers hardness of (Ti_65_(AlCrNbV)_35_)_100−x_B_x_ MEAs after thermomechanical treatment.

Composition	B0	B0.1	B0.2
As-rolled	419 ± 5	435 ± 3	456 ± 2
700 °C	388 ± 2	402 ± 3	431 ± 6
800 °C	347 ± 5	374 ± 3	399 ± 4
900 °C	322 ± 3	336 ± 4	353 ± 3

**Table 5:** Tensile mechanical properties of (Ti_65_(AlCrNbV)_35_)_100−x_B_x_ MEAs after thermomechanical treatment.

Processing	B0			B0.1			B0.2		
Mechanical Properties	Yield Strength	Ultimate Strength	Ductility	Yield Strength	Ultimate Strength	Ductility	Yield Strength	Ultimate Strength	Ductility
	(MPa)	(MPa)	(%)	(MPa)	(MPa)	(%)	(MPa)	(MPa)	(%)
As-rolled	1484	1547	4.5	1650	1713	4.4	N/A	N/A	N/A
700 °C	1249 ± 21	1367 ± 26	9.8 ± 0.7	1328 ± 11	1424 ± 23	9.1 ± 0.4	1351 ± 34	1446 ± 29	8.3 ± 1.2
800 °C	1098 ± 24	1321 ± 20	19.6 ± 1.2	1200 ± 24	1324 ± 14	10.5 ± 1.0	1275 ± 13	1415 ± 6	10.0 ± 1.2
900 °C	999 ± 3	1262 ± 8	30.2 ± 0.1	1050 ± 18	1295 ± 35	28.2 ± 1.3	1085 ± 14	1329 ± 19	24.6 ± 1.4

**Remark**: The as-rolled B0.2 MEA broke before passing the yielding point.

## Data Availability

Data are contained within the article.
